# Supplementary material for: Exploring healthcare staff experiences with a hybrid paper/digital health management information system and their perspectives on digitalization as an alternative – A Tanzanian qualitative case study on perinatal data
Source: PLoS One. 2025 Nov 7;20(11):e0335644. doi: 10.1371/journal.pone.0335644 (PMC12594368; doi:10.1371/journal.pone.0335644)
Supplement: Attachment — Submitted filename: PONE-D-_reviewer.pdf [file pone.0335644.s002.pdf]

Report

reviewer comment

Exploring healthcare staff experiences with a hybrid paper/digital health management information system and their perspectives on digitalization as an alternative a Tanzanian qualitative case study on perinatal data

Suggestions for Improvement:

1. Improve literature review, historical background and add future scope of study more effectively. To handle this issue, author can discuss some well related and recent references.
2. Language quality needs improvement.
3. The references should have a clear and unified format .
4. I have reviewed the paper. Presentation, the related literature and technical soundness are good. It presents good method. After made some additions to the paper, i recommend for publication.
5. Conclusion section should be enrich with some future remarks.
6. Some notations and equations are messed up or not clear. Please use a proper equation editor format to make them clearly visible and understandable
7. all symbols should be italic in math form.
8. All the notations and abbreviations should be checked carefully.
9. In Keywords section add more keywords.
10. Are these new results sharp and more accurate compared with the others?
11. Please check and if needed add all the missing punctuation or missing italics.
12. Some Theorem is not clear. Needs more discussion.
13. Please add (. and ,) in hole paper in suitable place.
14. I suggest adding some new relevant references to this topic.
    (a) New forms of contra-continuity in ideal topology spaces. Missouri Journal of Mathematical Sciences, 26(1):33–47, 2014.

(b) On almost e-$\mathcal{I}$-continuous functions NT, Demonstratio Mathematica 54 (1),2021, 168-177

(c) Degree of (L, M)-fuzzy semi-precontinuous and (L, M)-fuzzy semi-preirresolute functions,Demonstr. Math. 51 (1), 182197. Science

(d) Weak open sets on simple extension ideal topological space. Italian Journal of Pure and Applied Mathematics, 2014, (33), pp. 333344

(e) Multi granulation on nano soft topological space. Advances in Mathematics: Scientific Journal, 2020, 9(10), pp. 7711–7717.

(f) On topological groups via a-local functions, Applied General Topology, 2014, 15(1), pp. 3342

(g) New types of multifunctions in ideal topological spaces via e-J-open sets and $\delta\beta$-J-open sets, Boletim da Sociedade Paranaense de Matematica, 2016, 34(1), pp. 213223

Recommendation. I recommend accepting and publishing the paper after taking all the previous comments and observations and making some minor modifications mentioned above
