# Supplementary material for: Exploring healthcare staff experiences with a hybrid paper/digital health management information system and their perspectives on digitalization as an alternative – A Tanzanian qualitative case study on perinatal data
Source: PLoS One. 2025 Nov 7;20(11):e0335644. doi: 10.1371/journal.pone.0335644 (PMC12594368; doi:10.1371/journal.pone.0335644)
Supplement: Attachment — Submitted filename: Response to Reviewers.docx [file pone.0335644.s004.docx]

# 20^th^ September 2025

# Dear Editors and Reviewers,

# Thank you for the email we received detailing the reviewers’ comments on our manuscript. We thank the reviewers for the constructive, insightful and encouraging reviews.

# We have carefully reviewed the comments and revised the manuscript accordingly. The review process has supported us to improve our work, and we appreciate these contributions.

# Below we address the changes we have made in response to the two reviews. We present our responses in *italics*.

Yours sincerely,

Mary Cronin

School of Public Health,

University College Cork,

Ireland

______________________________________________________________________________

# **Reviewer #1: Report**

Suggestions for Improvement:

**1.** Improve literature review and add future scope of study more effectively. To handle this issue, author can discuss some well-related and recent references.

*Thank you for this suggestion. We have added more recent, related references. We have included a statement regarding future research in the Conclusion section.*

**2.** Language quality needs improvement.

*Thank you for this suggestion. We have revised and improved the standard of expression.*

**3.** The references should have a clear and unified format.

*Thank you for drawing this to our attention. We have revised and standardised the references using the Vancouver system.*

**4.** I have reviewed the paper. Presentation, the related literature and technical soundness are good. It presents good method. After made some additions to the paper, i recommend for publication.

*Noted with thanks.*

**5.** Conclusion section should be enrich with some future remarks.

*Thank you for this suggestion. We have included a statement regarding future research in the Conclusion section.*

**6.** Some notations and equations are messed up or not clear. Please use a proper equation editor format to make them clearly visible and under standable

*No notations or equations are present in the article as they are not relevant for a qualitative study.*

**7**. all symbols should be italic in math form.

*Thank you. We have included ‘n=29’ in italics in the article.*

**8.** All the notations and abbreviations should be checked carefully.

*Thank you for this suggestion. We have undertaken these checks and amended them accordingly.*

**9.** In Keywords section add more keywords.

*Thank you for this suggestion. More key words have been included.*

**10.** Are these new results sharp and more accurate compared with the others?

*Thank you for this suggestion. In the Findings section we have removed some quotes to reduce repetition. We have reported our findings accurately and compared / contrasted them with extant literature.*

**11.** Please check and if needed add all the missing punctuation or missing
Italics.

*Thank you for this suggestion. We have checked and addressed punctuation.*

**12**. Some Theorem is not clear. Needs more discussion.

*We have not included any Theorems as this is a qualitative study.*

**13.** Please add (. and ,) in hole paper in suitable place.

*Thank you for drawing this to our attention. We have checked and addressed punctuation.*

**14.** I suggest adding some new relevant references to this topic.
*Thank you for this suggestion. We have updated and added some other references. However, we do not believe the maths references (below) are appropriate for a qualitative study.*

(a) New forms of contra-continuity in ideal topology spaces. Missouri
Journal of Mathematical Sciences, 26(1):33–47, 2014.
(b) On almost e-I-continuous functions NT, Demonstratio Mathemat ica 54 (1),2021, 168-177
(c) Degree of (L, M)-fuzzy semi-precontinuous and (L, M)-fuzzy semi preirresolute functions,Demonstr. Math. 51 (1), 182197. Science
(d) Weak open sets on simple extension ideal topological space. Italian
Journal of Pure and Applied Mathematics, 2014, (33), pp. 333344
(e) Multi granulation on nano soft topological space. Advances in
Mathematics: Scientific Journal, 2020, 9(10), pp. 7711–7717.
(f) On topological groups via a-local functions, Applied General Topol ogy, 2014, 15(1), pp. 3342
(g) New types of multifunctions in ideal topological spaces via e-J open sets and δβ-J-open sets, Boletim da Sociedade Paranaense
de Matematica, 2016, 34(1), pp. 213223

Recommendation. I recommend accepting and publishing the paper after taking all the previous comments and observations and making some minor modifications mentioned above.

*Noted with thanks.*

*____________________________________________________________________________*

# **Reviewer #2: Report**

Reviewer #2: Thanks for the opportunity to this research manuscript that addressed a contemporary issue on healthcare staff experiences with a hybrid paper/digital Health Management Information System (HMIS) in Tanzania. The authors have done wonderful job.

*Noted with thanks.*

Here is my review of the qualitative study;

**Abstract**
Strengths:
Clear description of objectives, methodology (case study, semi-structured interviews), and analytical approach (thematic analysis). Findings are logically summarised, highlighting key challenges with the hybrid system and positive perceptions of digitalisation.

*Noted with thanks.*

Issues and Suggested Revisions:
The term "fully digital HMIS" appears repeatedly but could be more concise to improve readability.

*Thank you for this suggestion. We introduced the acronym DHMIS to improve conciseness.*

The sentence:
“In part, because of these challenges, there is a strongly positive attitude among healthcare staff towards adopting a fully digital system…”
Could be simplified for flow:
“These challenges contribute to the strongly positive attitude among healthcare staff towards adopting a fully digital system…”

*Thank you for this suggestion. Rewritten as suggested to simplify and improve flow.*

The recommendation section feels slightly compressed; splitting technical recommendations (training, integration, infrastructure) into clearer sentences would enhance readability.

*We added two sentences to expand and make this section clearer for the reader.*

**Introduction**
Strengths:
Well-structured with logical progression from global to national context and the use of recent WHO reports and region-specific literature strengthens the background.

*Noted with thanks.*

Issues and Suggested Revisions:
Grammatical inconsistency noted here:
“while simultaneously, many factors are known to impede or promote investment…”
The phrase "while simultaneously" is redundant; recommend:
“At the same time, many factors are known to impede or promote investment…”

*Thank you for this suggestion. Rewritten as suggested.*

Some citations are dated or rely heavily on prior Tanzanian studies, though recent sources (2023-2024) are included, which is commendable.

*We thought it useful to include some prior studies to reflect how this field has been evolving in Tanzania. Reviewer 1 has asked for some historical information, so we have retained them. However, we have included some additional, recent sources related to Tanzania and other countries.*

The sentence:
"Hesitancy to transition fully to digital systems has been reported among healthcare staff mainly due to perceived risks..."
Could benefit from clarification:
“Hesitancy to transition to fully digital systems has been reported among healthcare staff, often attributed to concerns regarding sustainability and funding…”

*Thank you for this suggestion. Rewritten as suggested.*

**Materials and Methods**
Strengths:
Provides a thorough explanation of research design, sampling, and context. Reflexivity statement reflects good qualitative research practice, and the ethical considerations are clearly outlined.

*Noted with thanks.*

Issues and Suggested Revisions:
Recruitment details imply purposive sampling but should explicitly state this.
Table 1 showing participant details is comprehensive, but one date seems inconsistent:
“NM 03 12/01/2022”
Should this be "12/01/2023" to align with other interview dates?

*Thank you for these suggestions. We have included a statement and reference regarding purposive sampling. The table contained 5 date errors. All have been amended to the correct dates.*

Under "Data Collection," this sentence could be clearer:
“Participants were provided with a small financial remuneration…”
Recommend specifying the amount or ethical justification for transparency.

*Thank you for this suggestion. We’ve specified the remuneration amount (500TSH).*

**Findings**
Strengths:
The thematic structure is logical and comprehensive. Direct participant quotes enrich the narrative and provide credibility.

*Noted with thanks.*

Issues and Suggested Revisions:
Some participant quotes are repetitive and could be trimmed for conciseness.

*We have removed some quotes to reduce repetition and shortened one quote. However, we think it is constructive to have evidence of shared perspectives across the different participant groups on an important point. We also believe there is a case to be made for including longer quotes in some instances, when there is detail and nuance included.*

This sentence:
“Regardless of the level of facility, or its public, faith-run or private status, the types of data collected and recorded are highly standardised.”
Could be improved for flow:
“The types of data collected and recorded are highly standardised across facilities, irrespective of their public, faith-based, or private status.”

*Thank you for drawing this to our attention. Rewritten as suggested.*

The transition between "Data Usage" and "Perceptions of a fully digitalised HMIS" could be made smoother with a bridging statement summarising challenges.

*Thank you for this suggestion. A linking statement has been added.*

**Discussion**
Strengths:
Findings are linked back to existing literature, enhancing validity. Discusses both clinical and policy-level implications.

*Noted with thanks.*

Issues and Suggested Revisions:
Repetition noted:
“These findings concur with those of Rumisha et al (1), Unkels et al(18), and Simba et al (13)…”

*The above statement has been rewritten with authors’ names removed and only reference numbers presented.*

Reference numbers are not consistently spaced; apply consistent formatting.

*Thank you for drawing this to our attention. Spacing has been fixed as suggested.*

This sentence:
“Several benefits of a fully digitised system were identified…”
Could be expanded briefly to include key examples for emphasis (e.g., improved data retrieval, workload reduction).

*Thank you for this suggestion. Rewritten as suggested.*

The claim:
“No negative attitudes were expressed, there was uniform support.”
Seems overly conclusive; recommend softening to:
“Participants expressed predominantly positive attitudes, with minimal reported reservations.”

*Thank you for drawing this to our attention. Rewritten as suggested.*

**Study Limitations**
Strengths:
Acknowledges limitations of sample size and geographic focus. Recognises potential for generalisability limitations.

*Noted with thanks.*

Issues and Suggested Revisions:
Contradiction:
“Constraints are mitigated by the diversity of contexts…”
Yet earlier states findings cannot be generalised. Suggest clarifying:
“While findings may not be generalised across Tanzania, the diversity of contexts within the sample enhances the transferability of insights.”

*Thank you for drawing this to our attention. Rewritten as suggested.*

Recommend acknowledging language translation as a limitation due to potential loss of nuance from Kiswahili to English.

*Thank you for drawing this to our attention. We have acknowledged this in the manuscript.*

**Conclusion**
Strengths:
Aligns with WHO strategies and national health policy goals, and conveys optimism for digital transition.

*Noted with thanks.*

Issues and Suggested Revisions:
This phrase:
“There is an opportunity again for Tanzania to take an evidence-based major leap forward…”
Is slightly informal for an academic paper; recommend:
“Tanzania has an opportunity to make a significant, evidence-informed advancement…”

*Thank you for drawing this to our attention. Rewritten as suggested.*

Final sentences could emphasise the role of continued research and evaluation post-implementation.

*Thank you for drawing this to our attention. We have rewritten the final sentences to include the role of continued research and evaluation post-implementation*.

**References**
Strengths:
Includes some recent and relevant sources (2023-2024).
Covers both global policy and Tanzanian-specific studies.

Issues and Suggested Revisions:
Some of the references can be upgraded using recent and relevant sources.

*We thought it useful to include some prior studies to reflect how this field has been evolving in Tanzania. Reviewer 1 has asked for some historical information, so we have retained them. However, we have included some additional, recent sources related to Tanzania and other countries.*

Inconsistent citation styles noted (e.g., missing author initials in some references).
Some hyperlinks (e.g., WHO document link) are incomplete or broken in the PDF format.

*We have revised and standardised the references using the Vancouver system. All hyperlinks are functioning at time of submission.*

Reference [11]:
“medRxiv preprint” Given this is a preprint, recommend caution in interpretation and explicitly noting preprint status in the discussion if cited for evidence.

*We have removed this reference and replaced it with a 2025 publication.*

**Summary of Major Recommendations:**

Improve conciseness and readability in the Abstract by simplifying complex sentences.
Correct minor grammatical inconsistencies in the Introduction (e.g., redundant phrases).
Explicitly state purposive sampling under Methods and verify date inconsistencies in Table 1.
Reduce repetitive participant quotes in Findings and ensure smooth section transitions.
Rephrase overly conclusive statements in the Discussion for academic caution.
Clarify contradictions regarding generalisability in Study Limitations.
Adopt more formal, precise language in the Conclusion.
Standardise and verify reference formatting, ensuring all hyperlinks are functional.

*Thank you for this summary. We believe that we have addressed these major recommendations.*
